# Visualization of cell-type dependent effects of anti-E2 antibody and interferon-gamma treatments on localization and expression of Broccoli aptamer-tagged alphavirus RNAs

**DOI:** 10.1038/s41598-020-61015-0

**Published:** 2020-03-24

**Authors:** Voraphoj Nilaratanakul, Debra A. Hauer, Diane E. Griffin

**Affiliations:** 1 W. Harry Feinstone Department of Molecular Microbiology and Immunology, Johns Hopkins Bloomberg School of Public Health, Baltimore, MD 21205 USA; 2 Cellular and Molecular Medicine Graduate Program, Johns Hopkins University School of Medicine, Baltimore, MD 21205 USA; 3 Present Address: Division of Infectious Diseases, Department of Medicine, Faculty of Medicine, Chulalongkorn University and King Chulalongkorn Memorial Hospital, Thai Red Cross Society, Bangkok, Thailand

**Keywords:** Alphaviruses, Molecular biology

## Abstract

Sindbis virus (SINV) is an alphavirus that causes age-dependent encephalomyelitis in mice. Within 7–8 days after infection infectious virus is cleared from neurons through the antiviral effects of antibody and interferon-gamma (IFNγ), but RNA persists. To better understand changes in viral RNA associated with immune-mediated clearance we developed recombinant strains of SINV that have genomic and subgenomic viral RNAs tagged with the Broccoli RNA aptamer that binds and activates a conditional fluorophore for live cell imaging of RNA. Treatment of SINV-Broccoli-infected cells with antibody to the SINV E2 glycoprotein had cell type-specific effects. In BHK cells, antibody increased levels of intracellular viral RNA and changed the primary location of genomic RNA from the perinuclear region to the plasma membrane without improving cell viability. In undifferentiated and differentiated AP7 (dAP7) neuronal cells, antibody treatment decreased levels of viral RNA. Occasional dAP7 cells escaped antibody-mediated clearance by not expressing cell surface E2 or binding antibody to the plasma membrane. IFNγ decreased viral RNA levels only in dAP7 cells and synergized with antibody for RNA clearance and improved cell survival. Therefore, analysis of aptamer-tagged SINV RNAs identified cell type- and neuronal maturation-dependent responses to immune mediators of virus clearance.

## Introduction

Sindbis virus (SINV) is a mosquito-borne enveloped plus-strand RNA virus that causes summertime outbreaks of rash and arthritis in humans and age-dependent encephalomyelitis in experimentally infected mice^1,2^. To initiate infection, the E2 glycoprotein attaches to the cellular receptor and E1 fuses the viral membrane with the cellular endosomal membrane to deliver the encapsidated genomic RNA into the cytoplasm. Upon cellular infection, the nonstructural replicase proteins are translated as a polyprotein from the viral genomic RNA. These proteins induce formation of spherules containing replication complexes at the plasma membrane where a full-length minus-strand copy of the RNA is transcribed. Subsequent processing of the replicase polyprotein converts the template specificity to plus strand synthesis of genomic and subgenomic RNAs. The abundant subgenomic RNA is the mRNA for translation of the structural capsid and E1 and E2 envelope viral proteins required for assembly of virions at the surface of the infected cell.

In tissue culture, highly permissive cells, such as baby hamster kidney (BHK) cells, replicate SINV to high titer and die by apoptosis within 24–48 h^3^. However, neurons, the primary target cells in the nervous system of mice with encephalomyelitis, show maturation-dependent susceptibility to infection. Immature dividing neurons replicate virus well and become apoptotic while mature differentiated non-dividing neurons restrict virus replication and can survive infection^4,5^. Furthermore, infected mature neurons can respond to treatment with immune mediators that decrease levels of intracellular virus^4,6–8^. *In vivo*, infectious SINV is non-cytolytically cleared from neurons in the brain and spinal cord within 7–8 days after infection by a combination of the site-specific effects of antibody to the E2 glycoprotein and interferon (IFN)-γ^7–10^. These immune mediators are produced within the central nervous system (CNS) by infiltrating lymphocytes^11,12^. Although infectious virus is cleared, viral RNA persists in the CNS and only slowly decreases over weeks to months and may never be completely eliminated^12–15^. This persistence of viral RNA is accompanied by continued residence and maturation of antibody-secreting B cells and IFNγ-producing T cells in the CNS^14,16,17^.

Identification of the mechanism(s) of antibody and IFNγ-mediated virus clearance depends on analysis of differentiated neurons that respond to treatment and are able to survive the acute phase of infection^7,18,19^. We have previously shown that cultured rat AP7 olfactory neurons provide an excellent model system for analyzing SINV replication in undifferentiated and differentiated neurons^6^. To visualize the regulation of viral RNA synthesis and degradation induced by host cell and immune factors we have developed strains of SINV with incorporated RNA aptamers that bind and activate a conditional fluorophore for excitation and fluorescence in live cells^20^.

In a recent advance over the imaging available with the Spinach2 aptamer^20^, multiple copies of the small bright Broccoli aptamer in a F30 scaffold^21,22^ were used to tag both genomic and subgenomic SINV RNAs (Nilaratanakul, Sci Rep, in press). These Broccoli-tagged SINVs replicate well in both neuronal and non-neuronal cells and viral RNA can be visualized in differentiated neurons, as well as more permissive BHK cells and undifferentiated neurons. In the current study, cells infected with SINVs with Broccoli-tagged genomic and subgenomic RNAs were evaluated for cell type-dependent changes in intracellular RNA in response to treatment with anti-SINV E2 antibody and/or IFNγ. In BHK cells, intracellular levels of viral genomic RNA were increased and re-localized by treatment with antibody while in both differentiated and undifferentiated AP7 neuronal cells, levels of viral RNA were decreased by antibody treatment. IFNγ showed synergism with antibody for improved viral RNA clearance and cell survival in differentiated AP7 cells, but had no effect on RNA clearance from BHK or undifferentiated AP7 cells.

## Results

### Infection of BHK cells and response to treatment with anti-E2 antibody

**Figure 1:**
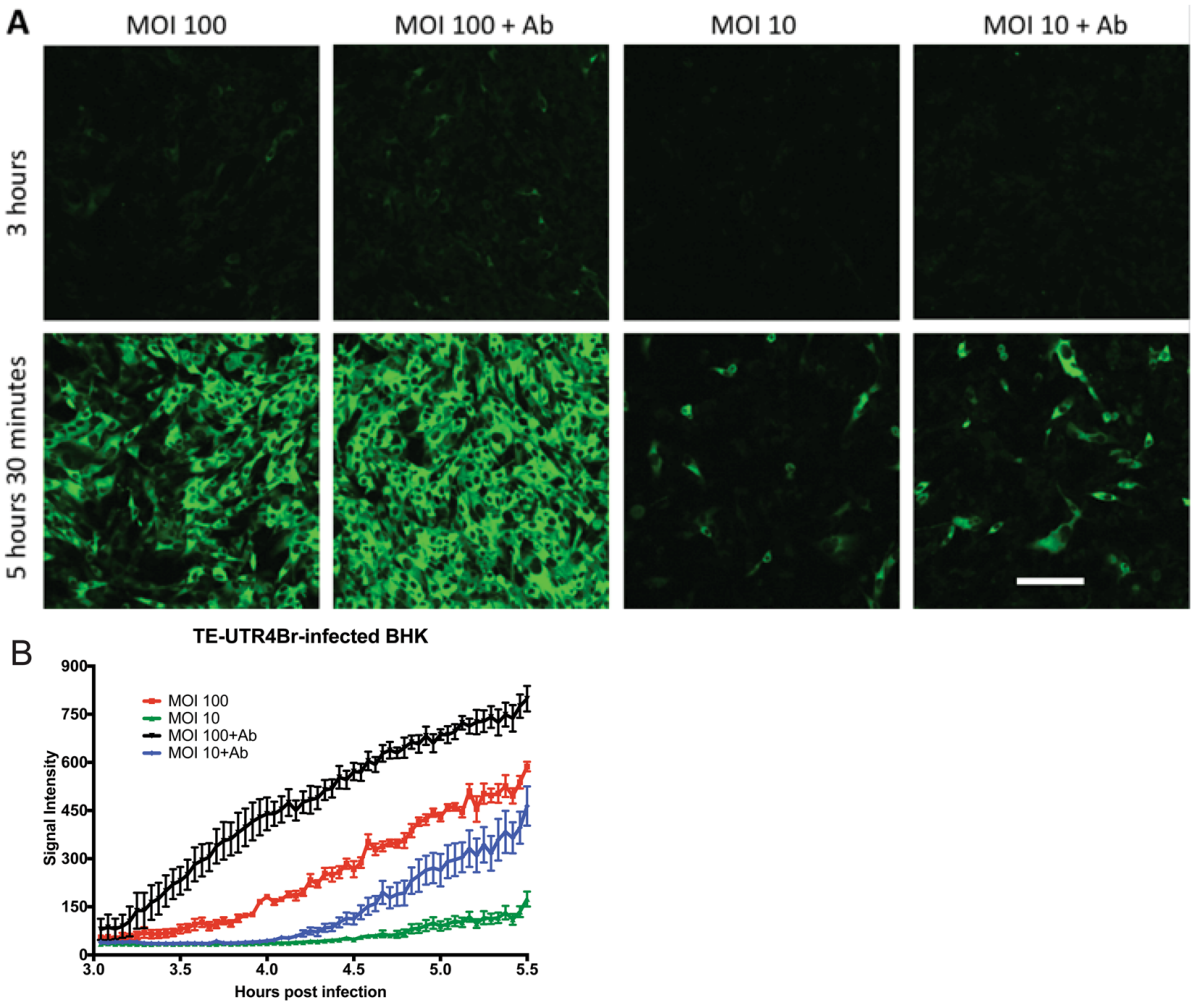
Intracellular SINV RNA levels in BHK cells are increased by higher MOI and by anti-E2 antibody treatment. Time-lapse live-cell imaging (**A**), objective lens 20X) of TE-UTR-4Br-infected BHK cells (3–5.5 h after infection) on the same settings shows the effects of multiplicity of infection (10 vs. 100) and anti-E2 [SV127] antibody treatment (5 μg/ml, 2 h after infection) on the accumulation of viral RNA (green). The graph (**B**) shows the average signal intensity of the 5 brightest cells from each group (1 frame = 150 seconds). Scale bar = 100 μm.

BHK cells are highly permissive for SINV replication and therefore were used for initial experiments to determine the effects of multiplicity of infection (MOI) and anti-E2 antibody treatment on visualization of Broccoli-tagged viral RNA. Time-lapse imaging from 3 to 5.5 h after infection was performed on cells infected at MOIs of 10 and 100 with TE-UTR-4Br, a recombinant SINV with 4 Broccoli aptamers incorporated into the 3′UTR of both genomic and subgenomic RNAs (Fig. 1) that replicates as well as wild-type virus in BHK cells. The levels of intracellular viral RNA after infection depended on the MOI (Fig. 1A,B) and increased with addition of anti-E2 antibody 2 h after infection (Fig. 1B).

**Figure 2:**
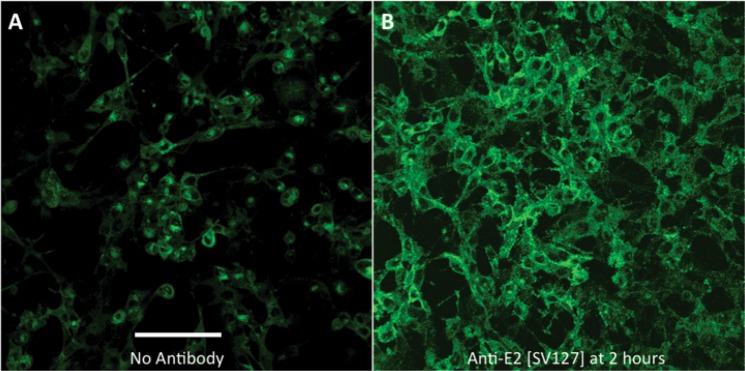
Effect of anti-E2 antibody treatment on viral RNA in BHK cells. Live-cell imaging (12 h after infection, objective lens 20X) to compare the effect of anti-E2 antibody treatment (5 μg/ml, 2 h after infection) (**B**) to no treatment (**A**) on genomic RNA levels and distribution in TEnsP3-4Br-infected BHK cells (MOI = 20). Without antibody treatment (**A**) the viral genomic RNA (green) is mainly present in the perinuclear area. With antibody treatment the RNA is accumulated at the plasma membrane (**B**). Scale bar = 100 μm.

To determine the effect of anti-E2 antibody treatment specifically on genomic RNA, BHK cells were infected with TE-nsP3–4Br, a recombinant SINV with 4 Broccoli aptamer copies incorporated into the hypervariable domain of the nsP3 gene in genomic RNA (Fig. 2). Imaging of cells 12 h after infection and 10 h after treatment with anti-E2 antibody showed that intracellular genomic RNA was increased and that the distribution was not perinuclear (Fig. 2A), but remained at the cell membrane (Fig. 2B) suggesting that antibody to E2 prevented migration of early replication complexes synthesizing genomic RNA from the cell surface^23,24^ and/or inhibited virus budding with accumulation of genomic RNA at the cell surface^25^.

### Infection of dAP7 cells and response to treatment with anti-E2 antibody and/or IFNγ

**Figure 3:**
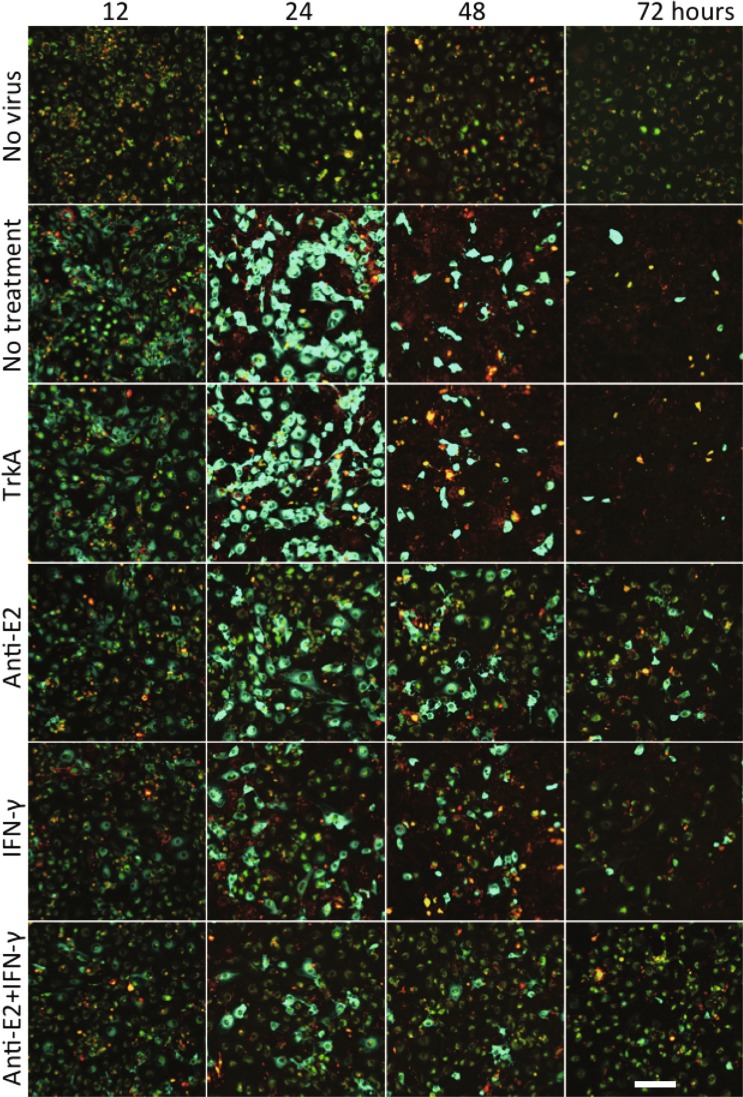
Antibody- and IFNγ-mediated SINV RNA clearance from dAP7 cells. Live-cell imaging (objective lens 20X, lambda mode) shows the effects of anti-E2 antibody and IFNγ on viral RNA clearance in TE-UTR-4Br-infected dAP7 cells (MOI = 20) at 12, 24, 48 and 72 h after infection (yellow – autofluorescence; green – Broccoli-DFHBI-1T; red – propidium iodide (dead cells)). All treatments (TrkA control antibody 5 μg/ml; anti-E2 5 μg/ml, rat IFNγ 500 U/ml) were applied 2 h after infection. Scale bar = 100 μm.

To determine the effect of anti-E2 antibody and IFNγ treatment on viral RNA in differentiated (d)AP7 neuronal cells, cells infected with TE-UTR-4Br were imaged from 12 to 72 h after infection using confocal lambda (spectral) mode microscopy that distinguishes the Broccoli signal from autofluorescence (Fig. 3). In contrast to BHK cells (Fig. 1), dAP7 cells treated 2 h after infection with anti-E2 antibody or IFNγ alone or in combination decreased intracellular viral RNA by 24 h and most cells cleared viral RNA by 72 h. Control anti-TrkA antibody had no effect on RNA clearance. By 72 h most of the untreated and anti-TrkA treated cells were dead and detached from the plate. Propidium iodide staining showed that treatment, especially the combination of anti-E2 antibody and IFNγ, also improved cell survival (Fig. 3**)**.

**Figure 4:**
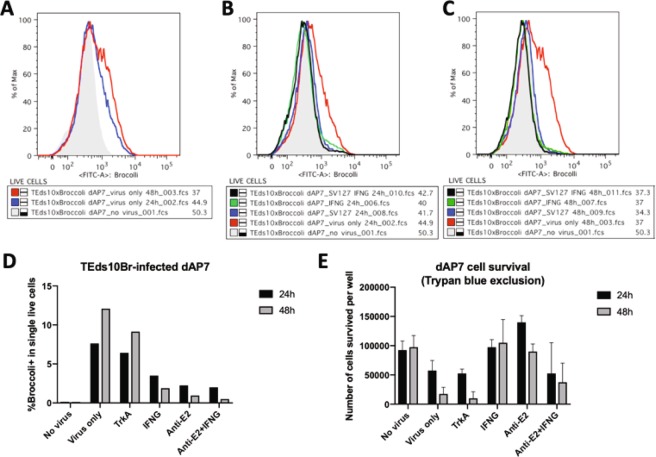
Effects of anti-E2 antibody and IFNγ on viral RNA clearance and cell survival in dAP7 cells. Flow cytometry histograms of the Broccoli-DFHBI-1T signal in dAP7 cells infected with TEds10Br (MOI = 20). (**A**) The signal intensity of infected cells at 24 h (blue) and 48 h (red) after infection in relationship to auto fluorescence (gray). (**B**,**C**) The effects of treatment at 2 h after infection with anti-E2 [SV127] antibody (blue) and/or IFNγ (black/green) in comparison to untreated cells (red) on the numbers of Broccoli-positive cells at 24 h (**B**) and 48 h (**C**). (**D**) Bar chart depicting the percentages of infected dAP7 cells with Broccoli signal greater than autofluorescence at 24 h (black) and 48 h (gray) after treatment with antibody and/or IFNγ. (**E**) Bar chart showing the effect of treatments on cell survival.

**Figure 5:**
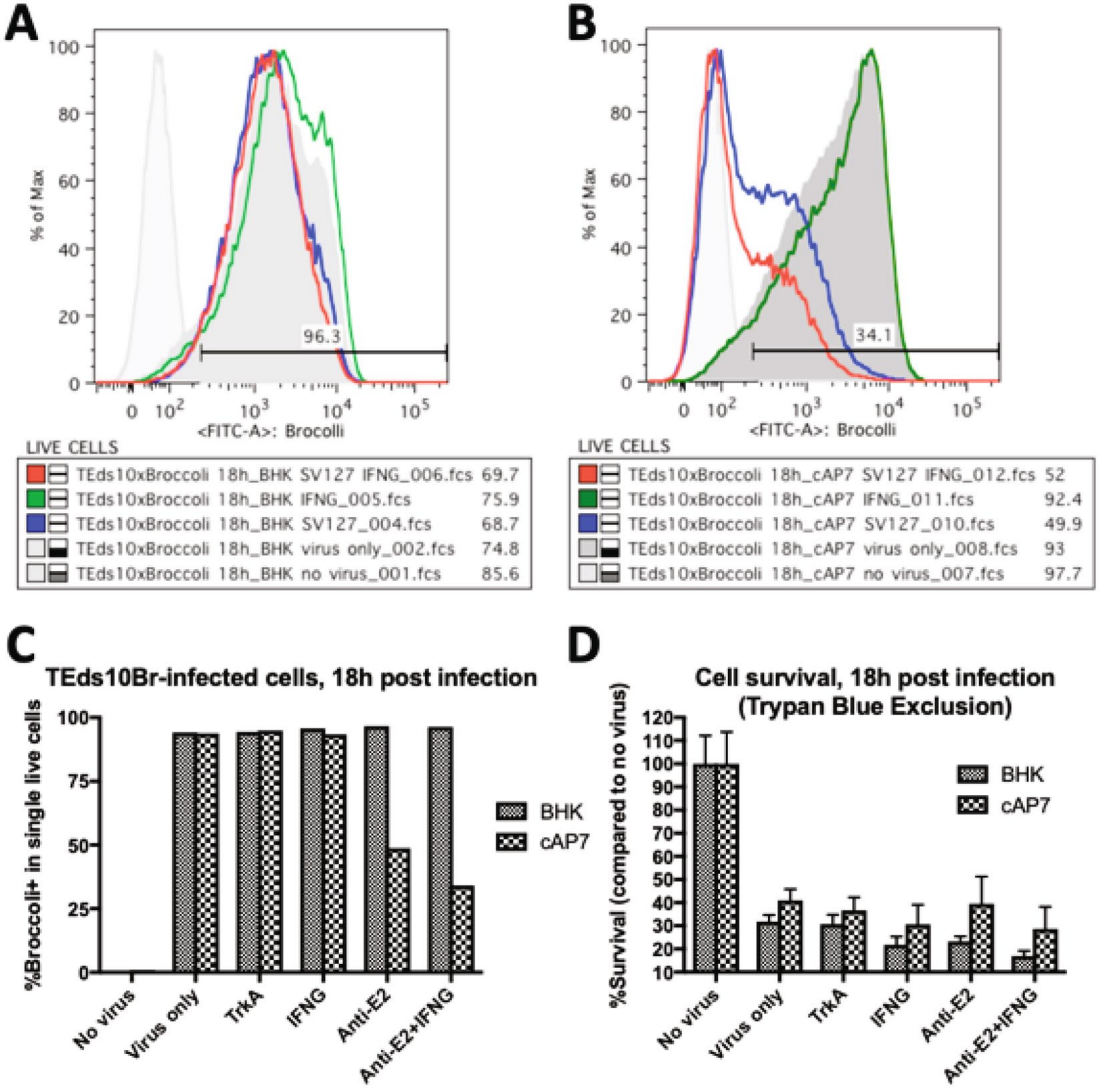
Effects of anti-E2 antibody and IFNγ on viral RNA clearance and cell survival in BHK and cAP7 cells. Flow cytometry histograms show the Broccoli-DFHBI-1T signal at 18 h after infection for BHK (**A**) and cAP7 (**B**) cells infected with TEds10Br (MOI = 5) and the effects of treatment with anti-E2 [SV127] antibody (blue) and/or IFNγ (green/red) at 2 h after infection compared to untreated cells (dark gray). (**C**) Bar chart of the numbers of Broccoli-positive BHK (gray) and cAP7 (hatched) cells after treatment with antibody and/or IFNγ. (**D**) Bar chart showing the effect of treatments on survival of infected BHK and cAP7 cells.

To confirm these observations, dAP7 cells infected with TEds-10Br, a recombinant double-subgenomic SINV with 6 Broccoli aptamers incorporated downstream of the second subgenomic promoter and another 4 Broccolis incorporated into the 3′UTR, were similarly treated with anti-E2 antibody, IFNγ or anti-TrkA antibody and analyzed by flow cytometry 24 and 48 h after infection (Fig. 4). Viral RNA in untreated infected cells increased between 24 and 48 h (Fig. 4A,D). Levels of viral RNA were decreased by treatment with either anti-E2 antibody or IFN-γ, but not by treatment with control anti-TrkA antibody (Fig. 4B,C). Effects of treatments on cell survival were analyzed by trypan blue exclusion and showed improvements both with anti-E2 antibody and IFNγ (Fig. 4E). Similar analyses of the effects of treatment on infected BHK cells showed that neither antibody nor IFNγ decreased viral RNA levels (Fig. 5A,C) or improved cell survival (Fig. 5D). Analysis of immature cycling (c) AP7 neuronal cells infected with TEds-10Br showed that antibody, but not IFNγ could mediate virus clearance in cAP7 cells (Fig. 5B,C) but cell survival was not improved (Fig. 5D). Therefore, BHK cells cannot restrict virus replication in response to immune mediators while both undifferentiated (cAP7) and differentiated (dAP7) neural cells decrease virus replication in response to treatment with anti-E2 antibody, but only dAP7 cells respond to IFNγ and have improved survival.

### Escape from anti-E2 antibody-mediated clearance

**Figure 6:**
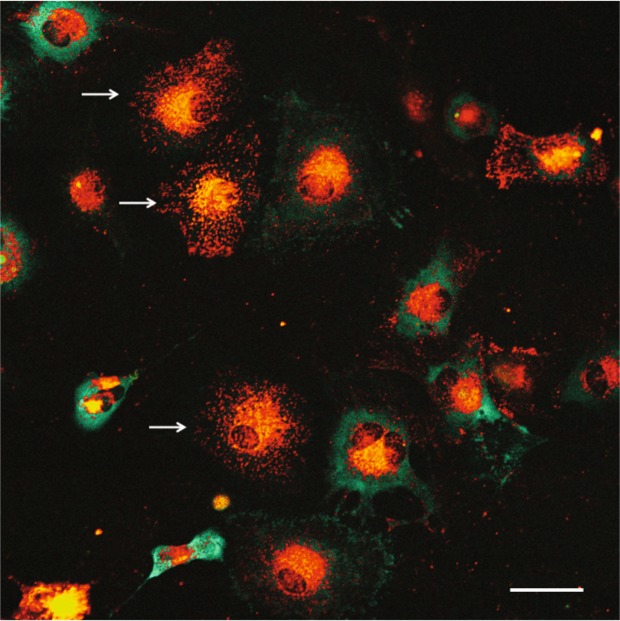
Inverse relationship between the amount of intracellular viral RNA and antibody on the cell membrane. Live-cell imaging (objective lens 20X, lambda mode) shows the amount of viral RNA (green) in TE-UTR-4Br-infected dAP7 (MOI = 20, 24 h after infection), treated with 1 μg/ml of SV127-Dylight594 at 2 h after infection. The cells that have antibody (red) bound to their membranes (white arrows) cleared the viral RNA, while the cells that lack antibody on their membrane did not. Scale bar = 50 μm.

We noted that viral RNA persisted in a few infected dAP7 cells despite treatment with anti-E2 antibody (Fig. 3). To determine how these cells differed from those that cleared viral RNA, TE-UTR-4Br-infected dAP7 cells were treated with anti-E2 antibody that had been conjugated with Dylight594 to identify infected cells that had antibody bound to the surface (Fig. 6). The amount of viral RNA was inversely correlated with the amount of anti-E2 bound to the cell surface.

**Figure 7:**
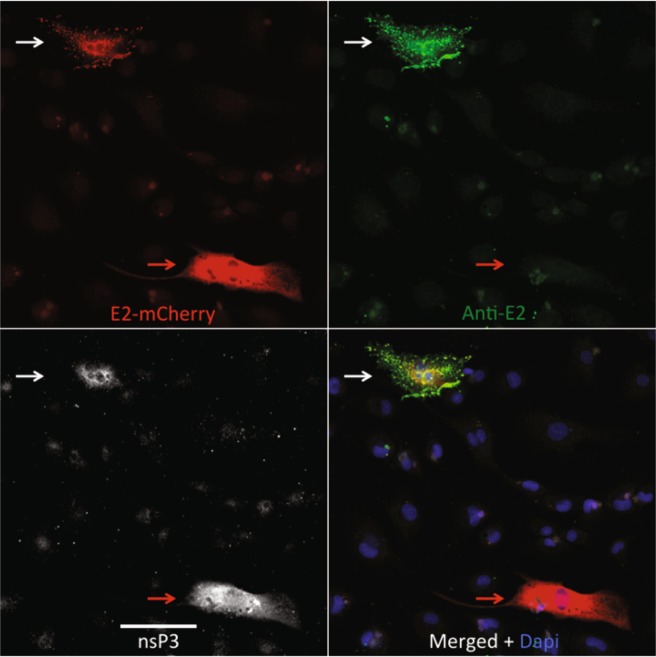
Lack of E2 envelope glycoprotein on the surface of some SINV-infected cells. dAP7 cells infected with SINV Toto E2-mCherry (MOI = 20), followed by treatment with anti-E2 [SV127, 5 μg/ml] 2 h after infection were fixed with 3.7% formaldehyde at 24 h were stained with anti-SINV nsP3 (white) and anti-mouse IgG (green). The viral E2 glycoprotein (red) is on the membrane of the cell that antibody (green) can bind (white arrows). In contrast, antibody cannot bind another cell that has only intracellular E2 (red arrows). Images are representative of multiple cells in the culture with similar staining. Scale bar = 100 μm.

To determine whether cells with little bound antibody were no longer synthesizing the E2 protein, dAP7 cells were infected with SINV expressing mCherry-tagged E2^26^ and treated with anti-E2 antibody (Fig. 7). Staining with antibody to nsP3 identified infected cells. Infected cells with bound anti-E2 antibody consistently expressed E2 on the plasma membrane while those without bound antibody had only intracellular E2 (Fig. 7). Therefore, some cells escaped antibody-mediated clearance of viral RNA by decreasing expression of cell-surface E2.

## Discussion

Understanding the mechanism(s) of noncytolytic virus clearance from neurons is important for identifying mechanisms of recovery from viral encephalomyelitis, developing treatments and preventing persistence. Previous studies have shown that the primary mechanism for SINV clearance from the CNS is antiviral antibody and that IFNγ can contribute to clearance, particularly from motor neurons in the spinal cord^8–11,17^. The current studies have used viruses with Broccoli aptamer-tagged RNAs to investigate the effects of treatments with antibody to the E2 glycoprotein and/or IFNγ on clearance of viral RNA from infected live cells. Effects on clearance of viral RNA were cell-type dependent (Table 1). Both anti-E2 antibody and IFNγ were effective in differentiated AP7 neurons, only anti E2 antibody was effective in immature neurons and neither was effective in BHK cells. Treatment improved survival only for differentiated neurons.

**Table 1:** Summary of treatment effects on different types of cells and mice.

Models	IFNγ- Ab-	IFNγ + Ab-	IFNγ- Ab+	IFNγ + Ab+
BHK	Dead in 18–24 h	←→RNA ←→survival	↑↑RNA ←→survival	↑↑RNA ←→survival
cAP7	Dead in 24–48 h	←→RNA ←→survival	↓RNA ←→survival	↓↓RNA ←→survival
dAP7	Dead in 48–72 h	↓RNA ↑survival	↓↓RNA ↑↑survival	↓↓↓RNA ↑↑↑survival
Mouse brain/ strain	Persistence/ SCID	Partial clearance/ μMT	Normal clearance/ IFNγ-/-	Normal clearance/ WT

The mechanism of antibody-mediated clearance is unclear. It is independent of immunoglobulin isotype and complement, but requires bivalent antibody suggesting that crosslinking of E2 on the cell surface induces clearance^7,8^. Studies of SINV-infected rat AT3-Bcl2 prostatic carcinoma cells showed that antibody treatment both increased RNA synthesis and inhibited virus budding leading to accumulation of nucleocapsids at the cell surface^25^. These effects of anti-E2 antibody are consistent with the current observations of increased RNA synthesis and antibody-induced accumulation of genomic RNA at the plasma membrane in BHK cells (Fig. 2). Increased viral RNA synthesis may reflect antibody-mediated reversal of virus-induced inhibition of host protein synthesis and prolonged synthesis as well as delayed processing of the replicase polyprotein resulting in sustained synthesis of negative strand RNA as observed in antibody-treated AT3-Bcl2 cells^25,27^. Because replication complex-associated spherules are formed at the plasma membrane^28^, antibody cross-linking at the cell surface may also prolong formation of new replication complexes. However, as shown with Spinach2-tagged viral RNAs in BHK cells infected at a low MOI, antibody-mediated inhibition of virus budding does not inhibit cell-to-cell spread of virus to uninfected cells^20^.

In contrast to the outcome of antibody-treated SINV-infected BHK cells, both undifferentiated and differentiated AP7 neuronal cells responded to antibody treatment with a reduction in intracellular viral RNA. Therefore, it is hypothesized that antibody crosslinking of E2 on the cell surface not only inhibits virus budding, but also induces an intracellular signaling cascade that reduces virus replication by affecting viral RNA synthesis and longevity. The current studies suggest that, in addition to inhibition of new viral RNA synthesis, antibody induces degradation of viral RNA in neural cells. Treatment also improved viability in dAP7 cells, but not cAP7 cells perhaps because of the intrinsic ability of mature neurons to restrict alphavirus replication that facilitates the response to antibody treatment^4,6^.

Some infected dAP7 cells had decreased expression of E2 on the cell surface and escaped from antibody-mediated clearance (Figs. 6, 7). In mice with SINV-induced encephalomyelitis, viral RNA persists for many months despite the presence within the CNS of high levels of antiviral antibody^12–14,16,29^. In SCID mice that cannot mount an adaptive immune response, passive treatment with antibody clears infectious virus, but there is reactivation in the brains of some animals after antibody has decayed suggesting persistence of full-length genomes^13^. However, the state of the viral RNA in immunologically competent mice with local synthesis of antibody within the CNS is not known. The current studies suggest that expression of viral proteins may be modulated to foster RNA persistence within infected cells in the presence of antibody.

Antibody-mediated clearance of viral RNA in dAP7 cells is complemented by the antiviral actions of IFNγ. IFNγ suppresses virus replication in differentiated neurons by signaling through the Jak/Stat pathway to phosphorylate Stat1 and Stat5^18,19^. IFNγ alone could clear viral RNA from dAP7 cells, but not cAP7 or BHK cells (Figs. 3–5). Previous studies with SINV-infected differentiated CSM14.1 neuronal cells in culture showed that treatment with IFNγ initially increased viral RNA synthesis followed by cessation of RNA synthesis consistent with the observed effects in dAP7 cells^18^. Activation of Stat transcription factors induces expression of many proteins with antiviral function that may be involved in suppression of viral replication, but the downstream effectors of clearance have not been identified. As demonstrated in these studies, the effects of IFNγ suppression of viral RNA are synergistic with the antiviral effects of antibody.

In summary, imaging of changes in viral RNA has enabled identification of cell type- and maturation-dependent changes in both amount and location of viral RNAs in response to treatment with immune mediators.

## Methods and Materials

### Cells

BHK-21 cells (ATCC) were cultured at 37 °C 5% CO_2_ in DMEM-10%FBS. AP7 odora cells (gift from Dale Hunter, Tufts University, Boston), derived from rat olfactory neurons and immortalized with a temperature-sensitive SV40 T antigen^30^, were either passaged as cycling cells (cAP7) in DMEM-10%FBS at 33 °C 7% CO_2_, or were differentiated for 7 days into neurons at 39 °C 5% CO_2_ (dAP7) by adding 1 μg/ml insulin, 100 μM ascorbic acid, and 20 μM dopamine (Sigma) into the medium as previously described^6^. Cells were infected with the recombinant viruses in DMEM-1%FBS at multiplicities of infection (MOI) of 5 to 100 as indicated in the figure legends.

### Viruses

A cDNA clone of the TE strain of SINV^31^ was the background for all aptamer-tagged recombinant viruses. Broccoli-tagged SINVs TE-UTR-4Br (4 Broccolis in the engineered *Sph1* site of the 3′UTR), TE-nsP3-4Br (4 Broccolis in the *Spe*1 site of the nsP3 gene) and TEds-10Br (TE-UTR-4Br plus 6 Broccolis after a second subgenomic promoter in the *BstE*II site) were constructed as previously described (Nilaratanakul *et al*., Sci Rep, in press)^20^. SINV Toto E2-mCherry was a gift from Richard Kuhn (Purdue University, West Lafayette, IN)^26^. Stocks were grown and assayed by plaque formation in BHK cells.

### Immunological reagents

For analysis of immune-mediated virus clearance mouse monoclonal IgG3 antibody against SINV E2 (SV127; 209^32^) was used. For antibody imaging, SV127 was conjugated with Dylight 594 (Thermo Scientific, ~5 dye molecules/IgG molecule) as previously described^20^. Infected cells were treated with 1–5 μg/ml of SV127 or 500 U/ml of rat IFNγ (PBL). Antibody to TrkA (Clone 315104, R&D Systems) at 1 μg/ml was used as a control. Infected cells were identified with a rabbit polyclonal antibody to SINV nsP3^33^ and CF633-conjugated anti rabbit IgG(H + L) (Biotium).

### Live-cell imaging

Cells were grown in 35 mm dishes, glass bottom 24-well plates (MatTek), or 8-well chamber cover glasses (Lab-Tek). Infected cells were imaged after replacing the culture media with phenol red-free imaging medium (Fluorobrite, Gibco) containing 20 μM DFHBI-1T (Lucerna), 25 mM HEPES (Gibco) at 37 °C for 30 min. The imaging medium plus 1% FBS, 1% PenStrep, and 2 mM glutamine was used for time-lapse imaging. Cell viability was assessed with 3 μM propidium iodide (Invitrogen). Autofluorescence was assessed relative to SINV TE (no aptamer)-infected cells and was minimized by adjusting the laser power and gain in each experiment. A laser scanning confocal microscope (Zeiss LSM780FCS, Axio Observer Z1, Zen Software) with 488 nm excitation was used to activate the Broccoli-DFHBI-1T complex. In dAP7 cells, the true signal was differentiated from autofluorescence with the lambda scan that captured an emission spectrum in each pixel. The Broccoli-DFHBI-1T signal had an emission peak at 507 nm and appeared green in lambda mode, while the autofluorescence signal had no peak, extended from green to far red in its spectrum, and appeared yellow. In time-lapse experiments, temperature and CO_2_ were kept at the same level as the culture condition for each cell type with a heated chamber at 24% humidity. ImageJ Software was used for image analysis.

### Flow cytometry

BHK, cAP7 and dAP7 cells with or without infection with SINV-Broccoli and antibody or IFNγ treatment were trypsinized, resuspended in imaging medium with DFHBI-1T and filtered through 70 μm nylon filter (Falcon). Data were collected on cells imaged in the GFP/FITC channel of a BD FACS Canto II flow cytometer. FlowJo (TreeStar) software was used for data analysis.
